# Supplementary material for: Transreplication Preference of the Tomato Leaf Curl Joydebpur Virus for a Noncognate Betasatellite through Iteron Resemblance on Nicotiana bethamiana
Source: Microorganisms. 2023 Dec 1;11(12):2907. doi: 10.3390/microorganisms11122907 (PMC10745424; doi:10.3390/microorganisms11122907)
Supplement: Supplementary file 1 [file microorganisms-11-02907-s001.zip › microorganisms-2720129-supplementary.pdf]

microorganisms

Article

Transreplication Preference of the Tomato Leaf Curl Joydebpur Virus for a Noncognate Betasatellite through *Iteron* Resemblance on *Nicotiana bethamiana*

Thuy T. B. Vo [1,†], I Gusti Ngurah Prabu Wira Sanjaya [1,†], Eui-Joon Kil [2], Aamir Lal [2], Phuong T. Ho [1], Bupi Nattanong [1], Marjia Tabassum [1], Muhammad Amir Qureshi [1], Taek-Kyun Lee [3,*] and Sukchan Lee [1,*]

[1] Department of Integrative Biotechnology, Sungkyunkwan University, Suwon 16419, Republic of Korea; bichthuy251188@gmail.com (T.T.B.V.); gusti.prabu20@gmail.com (I.G.N.P.W.S.); hophuongk59sinhhoc@gmail.com (P.T.H.); gum.bupi@gmail.com (N.B.); marjia39@g.skku.edu (M.T.); amirq303@gmail.com (M.A.Q.)
[2] Department of Plant Medicals, Andong National University, Andong 36729, Republic of Korea; viruskil@anu.ac.kr (E.-J.K.); aamirchaudhary43@gmail.com (A.L.)
[3] Risk Assessment Research Center, Korea Institute of Ocean Science & Technology, Geoje 53201, Republic of Korea
* Correspondence: tklee@kiost.ac.kr (T.-K.L.); cell4u@skku.edu (S.L.); Tel.: +82-31-290-7866 (S.L.)
† These authors contributed equally to this work.

Supplementary Materials

Table S1. Primer sets used to construct infectious clone.

Primer	Sequence 5'-3'	Target size
ToLCJoV-IC1-F	CTCGAGTGACTTGGTCAATCGGTGTC	1600 bp
ToLCJoV-IC1-R	CTGCAGCTCAGGCCGAGAAT	
ToLCJoV-IC2-F	CTGCAGTGATGGGTTCCCCT	1517 bp
ToLCJoV-IC2-R	AGATCTACACCTAAAACCGTGAACG	

Table S2. Sequence comparison of different ToLCJoVs compared with the ToLCJoV isolated in this study.

Acc No.	ToLCJoV	C1	C2	C3	C4	V1	V2	IR
MK330665	96.09%	94.66%	96.79%	98.02%	98.98%	96.63%	98.68%	96.63%
JN176565	92.03%	90.37%	94.32%	93.82%	90.17%	97.12%	99.33%	84.54%
JX311468	91.37%	92.1%	95.56%	93.33%	92.66%	92.38%	91.75%	87.76%
EU431116	98.41%	97.88%	99.01%	99.26%	98.64%	98.7%	98%	97.98%

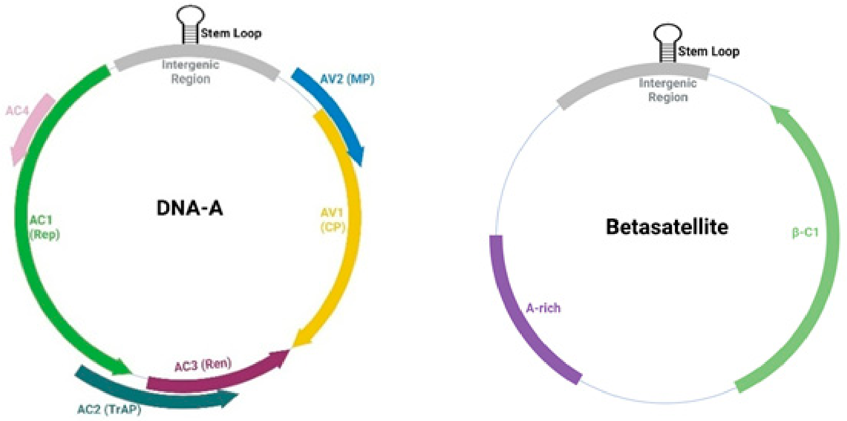

Figure S1. Genomic structure of ToLCJoV and ToLCBB. ToLCJoV including 6 ORFs encode for different viral proteins including Rep, TrAP, Ren, MP, CP and AC4. ToLCBB comprise β-C1 gene together with A-rich region.
